# Comprehensive transcriptomic analysis of hepatocellular Carcinoma: Uncovering shared and unique molecular signatures across diverse etiologies

**DOI:** 10.1016/j.bbrep.2025.102123

**Published:** 2025-06-30

**Authors:** Babak Khorsand, Nazanin Naderi, Seyedeh Sara Karimian, Maedeh Mohaghegh, Alireza Aghaahmadi, Seyedeh Negin Hadisadegh, Mina Owrang, Hamidreza Houri

**Affiliations:** a Department of Neurology, University of California, Irvine, CA, USA; b Department of Cell and Molecular Biology, Faculty of Life Science and Biotechnology, Shahid Beheshti University, Tehran, Iran; c Department of Oncology, University of Alberta, Canada; d Department of Biological Sciences, Islamic Azad University, North Tehran Branch, Tehran, Iran; e Department of Biology, Islamic Azad University of Central Tehran Branch, Tehran, Iran; f Department of Chemistry and Chemical Biology, Rutgers, The State University of New Jersey, Piscataway, NJ, USA; g Faculty of Medical Science, Sari Branch, Islamic Azad University, Sari, Iran; h Celiac Disease and Gluten Related Disorders Research Center, Research Institute for Gastroenterology and Liver Diseases, Shahid Beheshti University of Medical Sciences, Tehran, Iran; i Foodborne and Waterborne Diseases Research Center, Research Institute for Gastroenterology and Liver Diseases, Shahid Beheshti University of Medical Sciences, Tehran, Iran

**Keywords:** Differentially expressed genes, Hepatocellular carcinoma, Hepatitis B virus, Liver cirrhosis

## Abstract

Hepatocellular carcinoma (HCC) is a leading cause of cancer mortality, often diagnosed at advanced stages where treatment options are limited. This study undertakes a comprehensive meta-analysis of gene expression profiles from 19 independent datasets sourced from the Gene Expression Omnibus (GEO), encompassing a diverse range of HCC etiologies, including HBV and HCV infections, cirrhosis, and normal liver comparisons. Our analysis identified 125 genes consistently altered across all datasets (e.g., *CYP2C9*, *SLC22A1*, *RDH5*) that represent a pan-etiology HCC signature, implicating retinol metabolism and solute transport as key pathways in HCC pathogenesis. Notably, 14 HBV-specific differentially expressed genes (DEGs) (e.g., *ABCA8*, *GADD45B*) and 221 HCV-specific DEGs (e.g., *CDK1*, *CCNB1*) were identified, highlighting etiology-specific molecular signatures. Protein-protein interaction (PPI) networks revealed central hubs (e.g., CDK1, CCNE1, TYMS) involved in cell cycle dysregulation and metabolic reprogramming (Warburg effect). These findings provide a robust molecular framework for HCC subtyping and prioritize novel biomarkers and therapeutic targets for further validation. This resource advances the potential for personalized HCC diagnostics and therapies.

## Highlights

• 125 pan-etiology HCC genes (CYP2C9, SLC22A1) linked to retinol metabolism and solute transport.
• 14 HBV-specific and 221 HCV-specific DEGs reveal unique viral-driven HCC pathways.
• PPI hubs (CDK1, CCNE1, TYMS) drive cell cycle and metabolic dysregulation.
• Hub genes predict survival (CDK1 poor prognosis, CAT protective in HBV-HCC).

## 1 Introduction

Hepatocellular carcinoma (HCC) is the most common primary liver cancer, originating from hepatocytes. Globally, HCC remains a leading malignancy, ranking as the third most common cause of cancer mortality with over 900,000 new cases annually [1]. It constitutes over 80 % of liver cancer cases, with intrahepatic cholangiocarcinoma (iCCA) and other specified histologies accounting for 14.9 % and 5.1 % of cases, respectively [2]. HCC development is driven by a complex interplay of environmental and genetic factors. The typical onset of HCC occurs between ages 55 and 65, with variations across different populations, and it disproportionately affects males more than females [42]. Several risk factors are associated with HCC, including liver cirrhosis, chronic infections with hepatitis B virus (HBV) and hepatitis C virus (HCV), excessive alcohol consumption, exposure to aflatoxin B1, and nonalcoholic steatohepatitis (NASH) [3].

Among these, chronic HBV infection is a major etiological driver, contributing to approximately 50 % of HCC cases worldwide, with particularly high prevalence in East Asia and sub-Saharan Africa [4]. HBV promotes hepatocarcinogenesis through both direct and indirect mechanisms: (1) viral integration into the host genome disrupts tumor suppressor genes (e.g., *TP53*) or activates oncogenes (e.g., *TERT*), (2) the HBV X protein (HBx) dysregulates cellular signaling pathways (e.g., Wnt/β-catenin, NF-κB), and (3) chronic inflammation and immune-mediated liver injury drive cycles of necrosis, regeneration, and fibrosis [5]. Notably, HBV-related HCC often arises in non-cirrhotic livers, distinguishing it from HCV-related HCC, which typically requires cirrhosis as a precursor [6]. Additionally, HBV genotypes (e.g., genotype C) and viral load are linked to HCC progression, underscoring the need for etiology-specific biomarkers and therapeutic strategies [6].

Treatment options are limited to early-stage patients, with systemic therapies like immunotherapy showing variable responses [2,7,8]. The prognosis of HCC patients largely depends on the stage at diagnosis. Early detection is associated with improved overall survival, but most patients are diagnosed at advanced stages, precluding curative treatments like hepatic resection and liver transplantation, resulting in a 5-year survival rate of only 18 % [9]. Therefore, early detection and timely intervention are crucial for improving survival rates and quality of life for HCC patients. The use of biomarkers for predicting HCC, determining cancer stages, and targeting drug therapies holds significant potential for enhancing survival outcomes [10].

Several omics studies have identified key genetic and genomic markers of HCC, including the dysregulation of genes involved in cell cycle regulation, the Wnt/β-catenin pathway, cell adhesion molecules critical for cell-cell and cell-matrix interactions, and genes responsible for detoxification and immune response [9,[11], [12], [13]]. However, it remains unclear whether the integration of these gene dysregulations is also associated with the multiple etiologies of HCC and normal liver. More importantly, the identification of clinically significant transcriptomic changes in HCC across various etiologies reveals substantial variability in patient responses to immunotherapy. This variability complicates the development of effective immunotherapeutic strategies for different HCC subtypes. To address this knowledge gap and elucidate the molecular mechanisms of the identified cell types, this study aims to compile differentially expressed genes (DEGs) from bulk tissue studies to create a broadly applicable resource for HCC research. This comprehensive analysis encompasses 19 studies focused on gene expression profiling in HCC, with the goal of reporting DEGs.

## 2 Materials and methods

### 2.1 Benchmark dataset

**Fig. 1:**
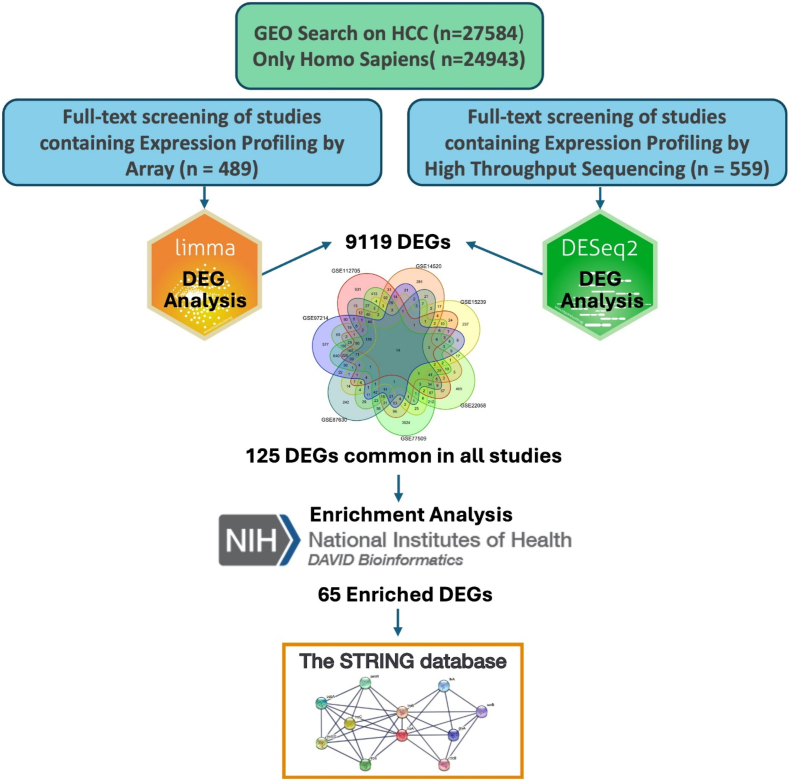
Workflow summarizing the systematic approach used to identify and characterize HCC-associated DEGs as follow: 1) GEO search for HCC (n = 27,584), filtered to include only Homo sapiens (n = 24,943). 2) Full-text screening of studies containing Expression Profiling by Array (n = 489) and Expression Profiling by High Throughput Sequencing (n = 559). 3) Differential gene expression analysis using limma for array-based studies and DESeq2 for sequencing-based studies. 4) Choosing common DEGs across all studies. 5) Enrichment analysis of the identified DEGs was performed using DAVID. 6) Construction of the protein–protein interaction (PPI) network using STRING.

A systematic workflow was followed to identify, screen, and analyze datasets (Fig. 1). This study utilized RNA sequencing and microarray datasets from the Gene Expression Omnibus (GEO) database, encompassing 19 distinct studies focused on gene expression profiling in HCC across various contexts, including early-stage, recurrent, and metastatic disease. The datasets cover a broad spectrum of sample types and clinical scenarios, providing a comprehensive basis for gene expression analysis in HCC.

### 2.2 Summary of included datasets

#### 2.2.1 Microarray datasets

##### 2.2.1.1 Samsung Medical Center microarray data (GSE36376)

Microarray data from 240 patients treated at Samsung Medical Center, Seoul, Korea (July 2000 to May 2006) were analyzed to identify gene expression signatures in HCC post-curative hepatectomy among young patients [14]. All patients had Child-Pugh Class A liver function. The analysis revealed 69 differentially expressed genes, mainly involved in cell cycle and cell division, differentiating patients aged ≤40 years from those >40 years.

##### 2.2.1.2 Genome-wide gene expression in HCC (GSE20140)

Microarray data from 287 HCC patients (tumor and adjacent non-tumor cirrhotic tissues) were analyzed, collected from four institutions within the HCC Genomic Consortium: Mount Sinai School of Medicine, New York; IRCCS Istituto Nazionale Tumori, Milan; Hospital Clinic, Barcelona; and Toranomon Hospital, Tokyo [15]. The study examined 22 gene signatures with prognostic value for early-stage HCC, predicting early and overall recurrence in conjunction with clinical and pathological findings.

##### 2.2.1.3 Pro-oncogenic pathways in HCC (GSE1898, GSE4024, GSE9829, GSE14520)

This analysis investigated pro-oncogenic pathways in primary tumors and adjacent non-malignant tissues using genome-wide mRNA expression profiles from 321 HCC patients [16]. Samples included 115 primary tumors and 52 adjacent tissues from the National Cancer Centre of Singapore/Sing Health Tissue Repository, and 206 HCC patients from the Liver Cancer Institute, Fudan University, Shanghai (GSE14520). The study identified 24 ribosomal genes as significant in both tumor and adjacent tissues, with *DKK1* serving as a biomarker for poor outcomes. The co-transcriptional signature of ribosome biogenesis genes suggests a novel predictive system.

##### 2.2.1.4 Genomic predictors for HCC recurrence (GSE12720, GSE15239, GSE39791, GSE22058, GSE14520)

This compilation includes microarray data from tumor and matched non-tumor tissues from 72 patients undergoing hepatectomy at Dongsan Medical Center, Keimyung University, Korea (cohort 1), and additional data from Queen Mary Hospital, University of Hong Kong (cohort 2, n = 96), and Fudan University, China (cohort 3, n = 228) [17]. The study identified a hepatocyte injury regeneration (HIR) signature of 233 unique genes, with four genes (*RALGDS*, *IER3*, *CEBPD*, *SLC2A3*) significantly associated with HCC recurrence and HBV. Network analysis revealed five upstream regulators: *NOTCH1*, *STAT3*, *PDX1*, *TP53*, and *RELA*, highlighting a notable interaction between *NOTCH1* and *STAT3*.

##### 2.2.1.5 Microarray data of carbon metabolism in HCC (GSE41804, GSE17548, GSE29721, GSE33006, GSE40873, GSE6222)

Two distinct microarray datasets were utilized to explore gene expression patterns in HCC and non-tumorous liver tissues [18]. Dataset Set-1 (E-MTAB-950) consisted of 120 HCC samples and 160 non-tumor samples, while Set-2 (GSE41804, GSE17548, GSE29721, GSE33006, GSE40873, GSE6222) included 60 HCC samples and 104 non-tumor samples. Additionally, RNA-seq data from three HCV-infected HCC patients (GSE81550) were analyzed to identify a 22-gene signature specifically associated with glycolysis regulation in HCC.

##### 2.2.1.6 Oncogenic markers in HCC subgroups (GSE14520, GSE1898, and GSE4024)

Gene expression data from 380 HCC patients identified the oncoprotein YY1AP1 as a critical factor in the EpCAM + AFP + HCC subtype [19]. The involvement of YY1AP1 in chromatin topology and stem cell regulation underscores its potential as a therapeutic target in this aggressive HCC subgroup.

#### 2.2.2 RNA sequencing datasets

##### 2.2.2.1 RNA-sequencing data from Seoul National University Hospital (GSE77509)

This dataset comprises RNA sequencing data from 62 early malignant HCC tumor samples, including 38 patients who underwent surgical resection, alongside 15 normal and 47 adjacent non-tumor samples from patients with HCC or chronic liver disease at Seoul National University Hospital [20]. The study aimed to elucidate transcriptomic profiles during HCC development, revealing a comprehensive immune landscape and dynamic shifts in transcriptomes across 22 major immune cell types from non-tumorous to early malignant stages. Regulatory T cell (Treg) infiltration was noted, with a potential association between *CYB561* gene expression and adverse outcomes such as angioinvasion or tumor relapse in patients undergoing total hepatectomy.

##### 2.2.2.2 HBV-associated early-stage HCC transcriptome (GSE124535)

This dataset includes RNA sequencing data from 35 HBV-associated early-stage HCC tissues (29 males and 6 females, average age: 55) and 35 healthy tissues collected from Zhongshan Hospital, Fudan University, and Cancer Hospital & Institute, Peking University [21]. The analysis identified eight hub genes (upregulated: *AURKB*, *CDK1*, *CDC20*, *CCNB1*, *CCNA2*; downregulated: *EHHADH*, *APOA1*, *UBB*) and key signaling pathways, including metabolic pathways, complement and coagulation cascades, PPAR signaling, and fatty acid degradation. Notably, *CDK1*, *CDC20*, *CCNB1*, and *CCNA2* were significantly involved in the cell cycle and p53 signaling pathway.

##### 2.2.2.3 Differential gene expression analysis (GSE89377 and GSE114564)

The microarray dataset GSE89377, containing 85 HCC tissue samples (24 recurrence and 8 non-recurrence), and the RNA-seq dataset GSE114564, including 53 HCC tissue samples (21 recurrence and 32 non-recurrence), were analyzed to identify novel gene signatures associated with HCC recurrence [22]. A total of 2385 and 5927 DEGs were found in the two datasets, respectively, with 981 common DEGs identified via Venn diagram. Gene ontology (GO) analysis linked these genes to biological processes such as the Wnt signaling pathway, angiogenesis, and blood coagulation. Gene set enrichment analysis highlighted candidate genes, including *CETN2*, *HMGA1*, *MPZL1*, *RACGAP1*, and *SNRPB* as predictive markers for HCC recurrence. Additionally, *HMGA1* and *RACGAP1* emerged as distinct prognostic indicators.

##### 2.2.2.4 Transcriptome sequencing analysis in HCC from Fujian Provincial Hospital (GSE97214)

The discovery analysis used RNA-seq data from 9 matched pairs (GSE97214), while the full Fujian cohort (n = 63) provided validation samples as per Huang et al.

RNA-seq data from 63 HCC patients at Fujian Provincial Hospital, China, were analyzed to compare HCC tumors and adjacent non-tumorous tissues [23]. The study identified 943 DEGs, with 690 upregulated and 1253 downregulated genes involved in pathways such as the cell cycle, DNA replication, p53 signaling, and coagulation cascades. Seven fusion genes were detected, with CRYL1-IFT88 recurring in approximately 9.52 % of cases, potentially contributing to HCC progression by reducing the tumor suppressor function of IFT88.

##### 2.2.2.5 RNA-seq data from advanced-stage HBsAg positive HCC patients (GSE128274)

The dataset contains paired tumor/paratumor samples from 4 HCC patients, profiling lncRNA (n = 1832), circRNA (n = 945), and miRNA (n = 328) expression via RNA-seq [24]. All patients were HBsAg positive and had not received anti-tumor treatment prior to surgery. Differential expression was noted in lncRNAs, miRNAs, and circRNAs, with volcano plots identifying significant differentials in mRNAs, lncRNAs, miRNAs, and circRNAs, highlighting gene-specific hypo-editing and hyper-editing patterns in HCC.

##### 2.2.2.6 IsomiR-21-5p in liver cancer progression (GSE114564)

RNA sequencing data of 15 Normal liver, 20 Chronic hepatitis, 10 Liver Cirrhosis, 10 Dysplastic nodule, 18 Early HCC, and 45 Advanced HCC samples of 86 patients [25].

##### 2.2.2.7 Lipid metabolism reprogramming in HCC (GSE140463 and GSE140243)

RNA-seq data from seven patients with HCC undergoing liver transplantation at Addenbrooke's Hospital were analyzed to study lipid metabolism reprogramming in cirrhotic fatty liver [26]. An integrated systems biology approach revealed a positive correlation between MUFA-PC levels and genes involved in lipogenesis and PC synthesis, highlighting the significance of MUFA-PC in HCC proliferation.

##### 2.2.2.8 Metabolism-related genes in HCC carcinogenesis (GSE77314)

RNA sequencing data from 50 HCC patients, encompassing both tumor and peri-tumoral tissues, were examined to identify DEGs across different metastasis stages [27]. The analysis revealed 8900 DEGs in early metastasis stages and 1789 DEGs in advanced metastasis stages. Notably, the gene DMGDH was found to be downregulated, suggesting its potential role as a predictive marker for HCC and its involvement in inhibiting metastasis via the Akt signaling pathway.

##### 2.2.2.9 DNA methylation patterns in HCV-associated HCC (GSE82178)

RNA-seq data from paired tumor (n = 9) and non-tumor tissues from HCV-infected (n = 10) and uninfected individuals (n = 11) were analyzed to identify distinct DNA methylation patterns associated with HCC development [28]. The study highlighted the addition of DNA methylation targeted to candidate enhancers in liver cells, particularly enriched at the binding sites of *FOXA1*, *FOXA2*, and *HNF4A* transcription factors. These methylation patterns were implicated in the regulation of genes related to liver cancer and stem cell biology.

##### 2.2.2.10 RNA sequencing data indicated prognostic subtypes in HCC (GSE87630)

In a comprehensive multi-omics analysis, RNA-seq data identified three prognostic subtypes of HCC, designated as iCl1, iCl2, and iCl3, with the iCl1 subtype exhibiting the poorest prognosis [29]. The study linked deviations in *CNVcor* and *METcor* genes to unfavorable clinical outcomes, with CA9 emerging as a key differentially expressed gene in more invasive tumor phenotypes.

##### 2.2.2.11 RNA sequencing data of ribosome profiling in HCC (GSE112705)

Ribosome profiling data from 10 HCC patients were analyzed, revealing dysregulated translation processes in HCC at sub-codon resolution [30]. The study identified significant variations in translation efficiency and frame usage, which may have important implications for understanding the molecular mechanisms underpinning tumor biology.

##### 2.2.2.12 Comprehensive transcriptome profiling in HCC (GSE105130)

RNA sequencing data from 25 HCC patients, predominantly HBV-positive, were analyzed to identify 53,224 transcripts with significant differential expression between tumor and non-tumor tissues [31]. Pathway analysis indicated enrichment in cell cycle regulation, apoptosis, and DNA repair pathways, with key regulators such as *CDK1* and *TP73* playing crucial roles in HCC progression.

### 2.3 Data analysis pipeline and statistical analysis

For microarray datasets (CEL files), raw data were processed using the Robust Multi-array Average (RMA) algorithm for background correction, quantile normalization, and summarization, as implemented in the affy package in R. For RNA-sequencing datasets (FASTQ files), raw reads underwent quality control using FastQC, followed by alignment to the human reference genome (GRCh38) using the STAR aligner. Gene-level quantification was performed using featureCounts.

Differential expression analysis was conducted using the DESeq2 package. The Wald test was employed to identify differentially expressed genes, and p-values were adjusted for multiple testing using the Benjamini-Hochberg method to control the false discovery rate. Microarray DEGs were identified using the limma R package (v3.54.1) with thresholds of |log2 fold change (FC)| ≥ 1 and adjusted *p*-value <0.05 (Benjamini-Hochberg correction). Functional enrichment analysis of the DEGs common across all studies was performed using DAVID Bioinformatics Resources. The significantly enriched DEGs were then used to construct a protein–protein interaction (PPI) network through the STRING database to investigate the molecular interactions underlying HCC further and to elucidate the potential mechanisms that distinguish HCC from other liver conditions. The PPI network constructed in STRING was exported to Cytoscape, and hub genes were identified using the CytoHubba plugin by selecting those with the highest degree of connectivity.

### 2.4 Principal component analysis (PCA)

For each study, we first selected the subset of genes consistently identified as DEGs across all datasets. Principal Component Regression (PCR) was then performed separately on the DEGs from each study. To determine the optimal number of principal components (PCs), we applied cross-validation (CV) and selected the smallest number of PCs for which the mean squared prediction error (MSEP) was within one standard error of the minimum, ensuring a balance between model performance and interpretability. Gene contributions to each principal component were quantified using the component loadings, where a higher absolute loading indicates greater influence of the gene on that component. To derive an overall gene importance score, we summed the absolute loading values across the selected top components for each gene. These scores were normalized within each study, and the mean importance score across all studies was used as the final gene importance measure. Gene importance scores for all genes are provided in Supplementary File 1.

## 3 Results

**Fig. 2:**
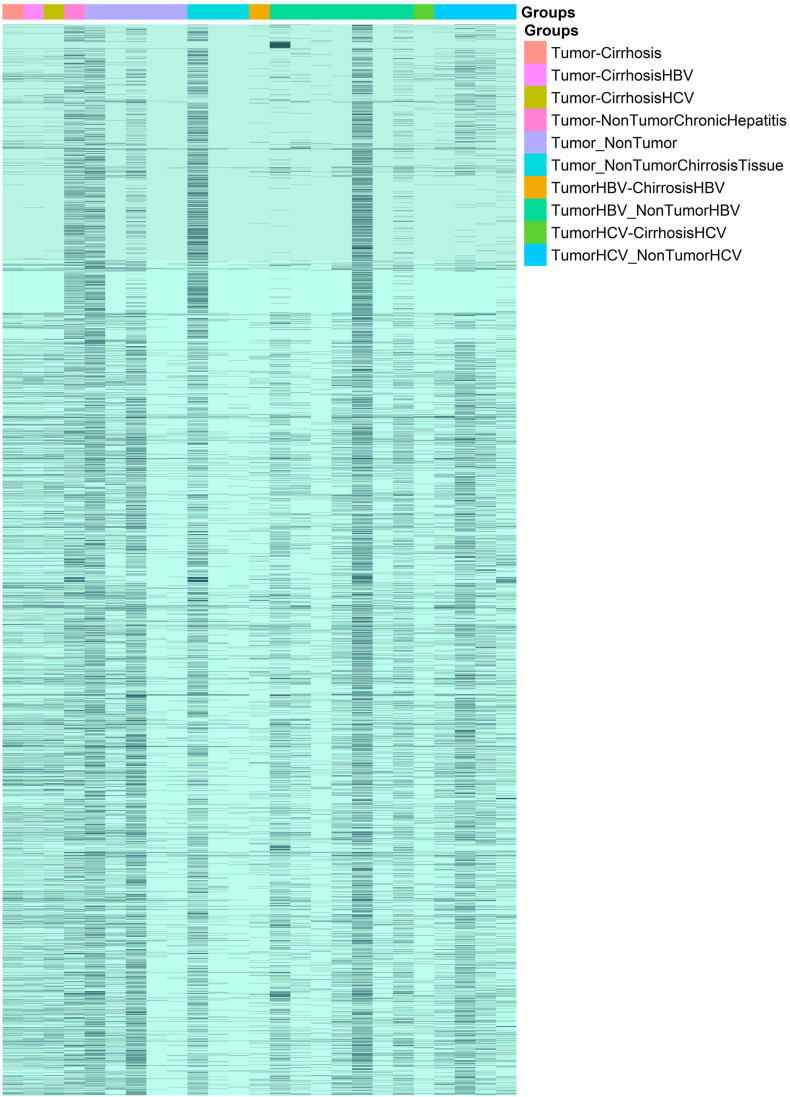
Heatmap illustrating the identified DEGs of HCC (Tumor) compared to various liver conditions. Color scale represents log2 fold change values, with dark blue indicating significant differential expression (|log2FC| ≥ 1, FDR <0.05) and light blue indicating no significant change (|log2FC| < 1 or FDR ≥0.05).

Our comprehensive analysis identified common DEGs in HCC by examining 19 distinct dataset groups comparing HCC tissues against various liver conditions. Key comparisons included: (1) HBV-infected HCC vs non-cancerous HBV tissue (7 datasets), (2) HCC vs normal liver (5 datasets), (3) HCV-infected HCC vs non-cancerous HCV tissue (4 datasets), and multiple cirrhosis-related comparisons (3-1 datasets each). This systematic approach enabled us to detect consistently altered molecular signatures across different HCC etiologies and disease stages. The resulting DEG patterns, visualized in the heatmap of Fig. 2, provide crucial insights into shared pathogenic mechanisms underlying HCC development and progression, regardless of etiology.

### 3.1 125 conserved DEGs robustly differentiate HCC from normal liver tissue

**Fig. 3:**
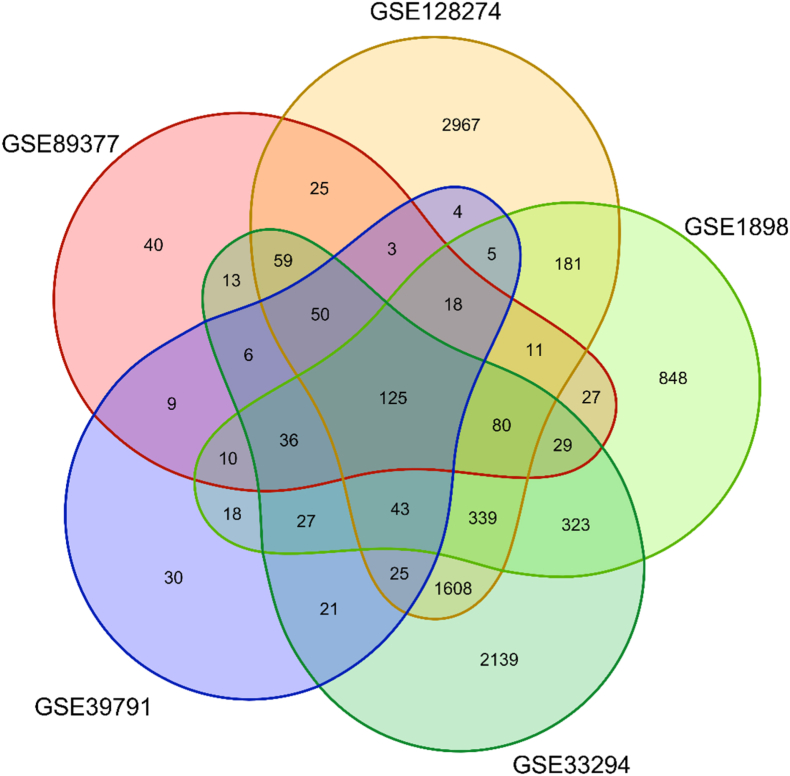
A Venn diagram that visually represents the overlap of DEGs among five datasets comparing HCC tissue with normal liver tissue.

To uncover the DEG profiles distinguishing HCC from normal liver tissue, we analyzed data from five independent datasets. Specifically, we identified 627 DEGs in GSE89377 (RNA-seq data), 5543 DEGs in GSE128274 (RNA-seq data), 2210 DEGs in GSE1898 (microarray data), 4923 DEGs in GSE33294 (RNA-seq data), and 477 DEGs in GSE39791 (microarray data). Comprehensive details of the identified DEGs, along with their expression patterns across these datasets, are provided in Supplementary File 2. Notably, 125 genes demonstrated consistent differential expression (FDR <0.05, same log2FC direction) across all five datasets, representing a pan-etiology HCC signature. These include cell cycle regulators (∗CDK1, CCNE1) and metabolic enzymes (CYP2C9), with prior HCC associations in literature, strongly supporting their utility as core diagnostic biomarkers. The full list of these common DEGs is presented in Supplementary Table S1. Moreover, Fig. 3 provides a Venn diagram that visually represents the overlap of DEGs among the datasets comparing HCC tissue with normal liver tissue.

### 3.2 14 HBV-specific DEGs reveal unique molecular drivers of HBV-related hepatocarcinogenesis

To identify DEGs between HCC in HBV-infected patients and their non-cancerous counterparts, we analyze 2860 DEGs in GSE112705, 1672 DEGs in GSE14520, 608 DEGs in GSE15239, 2408 DEGs in GSE22058, 6784 DEGs in GSE77509, 1164 DEGs in GSE87630, and 2804 DEGs in GSE97214. This comprehensive approach led to the identification of a total of 10,018 DEGs, encompassing 6280 upregulated genes and 3738 downregulated genes. A detailed analysis of the expression profiles and the distribution of these DEGs across the datasets is provided in Supplementary File 3.

Notably, 14 genes were consistently differentially expressed across all seven datasets, indicating their potential as robust biomarkers for distinguishing HCC in HBV-infected patients from non-cancerous HBV-infected tissue (Supplementary Table S2). These key genes include ATP-binding cassette subfamily A member 8 (*ABCA8*), catalase (*CAT*), C-C motif chemokine ligand 20 (*CCL20*), *F11*, GABA type A receptor-associated protein-like 1 (*GABARAPL1*), growth arrest and DNA damage-inducible beta (*GADD45B*), GTP cyclohydrolase 1 (*GCH1*), metallothionein 1G (*MT1G*), metallothionein 1 M (*MT1M*), regulator of calcineurin 1 (*RCAN1*), Rho family GTPase 3 (*RND3*), S100 calcium-binding protein P (*S100P*), suppressor of cytokine signaling 2 (*SOCS2*), and ZFP36 ring finger protein (*ZFP36*).

**Fig. 4:**
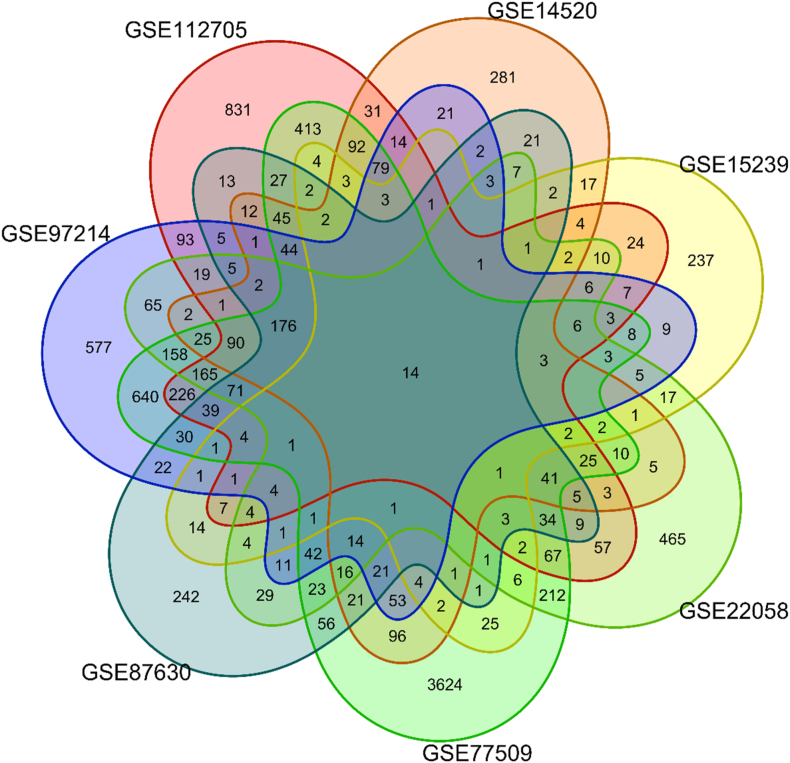
Venn diagram visualizing the intersection of DEGs among the seven datasets, highlighting the genes consistently associated with HCC in HBV-infected patients compared to HBV-infected patients without HCC.

Additionally, 182 and 288 DEGs were found to be common across six and five datasets, respectively. The Venn diagram in Fig. 4 visualizes the intersection of DEGs among the seven datasets, highlighting the genes consistently associated with HCC in HBV-infected patients.

### 3.3 HCV-associated HCC shows distinct metabolic reprogramming via 221 conserved DEGs

**Fig. 5:**
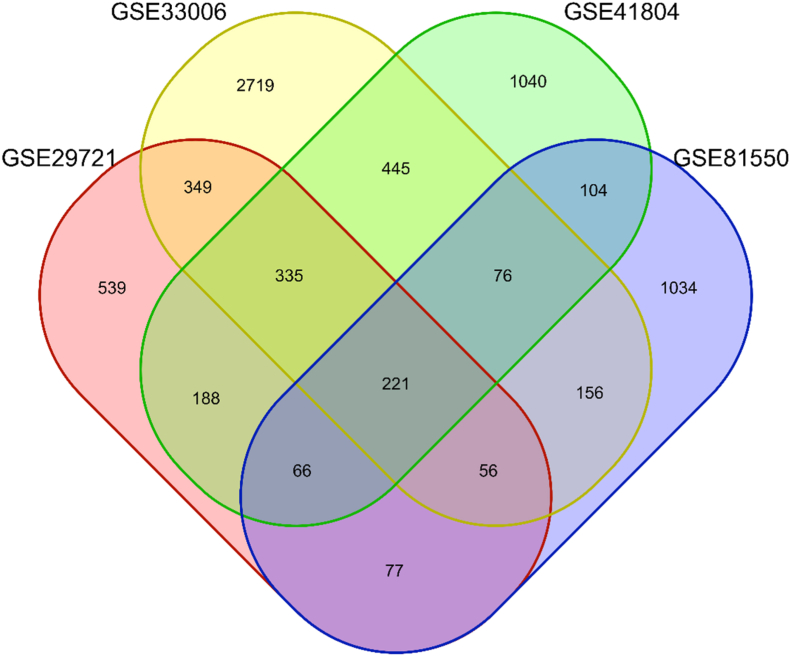
Venn diagram visualizing the intersection of DEGs among the seven datasets, highlighting the genes consistently associated with HCC in HCV-infected patients compared to HCV-infected patients without HCC.

To identify the DEGs between HCC in HCV-infected patients and their non-cancerous counterparts, we conducted an analysis of the following datasets: GSE29721 (2403 DEGs), GSE33006 (5309 DEGs), GSE41804 (3252 DEGs), and GSE81550 (1790 DEGs). Our comprehensive examination resulted in the identification of 7403 DEGs in total, comprising 3395 upregulated and 4008 downregulated genes. The detailed expression profiles and distribution of these DEGs across the datasets analyzing HCC in HCV-infected patients and their non-cancerous counterparts are available in Supplementary File 4. Importantly, 220 genes were consistently differentially expressed across all four datasets, underscoring their potential as reliable biomarkers for distinguishing HCC in HCV-infected patients from non-cancerous HCV-infected tissue (as listed in Supplementary Table S3). Fig. 5 illustrates the Venn diagram depicting the intersection of DEGs among the datasets, highlighting the genes consistently associated with HCC in HCV-infected patients.

### 3.4 164 DEGs uniquely differentiate HCC from cirrhosis, offering early detection biomarkers

**Fig. 6:**
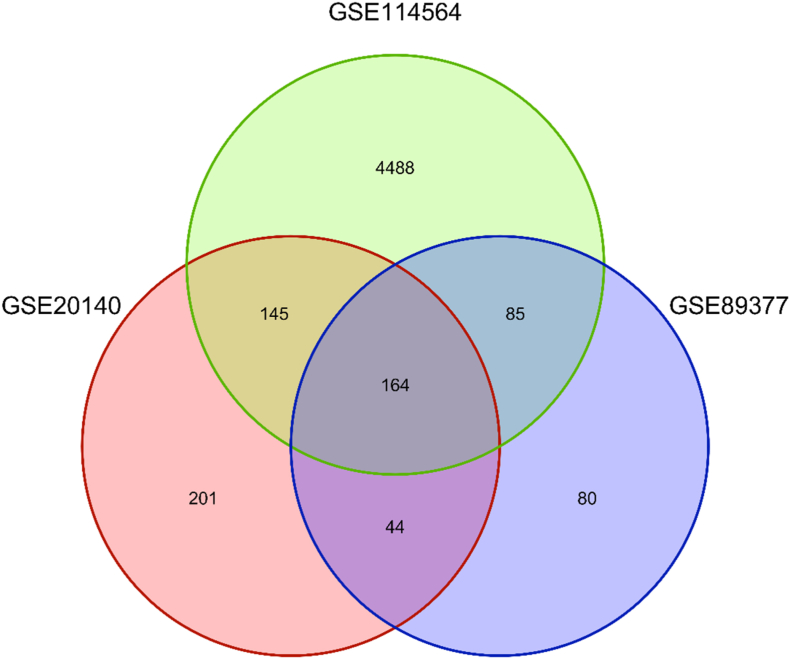
Venn diagram illustrating the overlap of DEGs among three datasets, emphasizing the genes consistently associated with HCC in comparison to cirrhosis.

To distinguish DEGs between HCC and cirrhosis, we analyzed data from the following datasets: GSE20140 (648 DEGs), GSE114564 (4882 DEGs), and GSE89377 (431 DEGs). This comprehensive analysis identified a total of 5207 DEGs, including 3087 upregulated and 2120 downregulated genes. The detailed expression profiles and distribution of these DEGs across the datasets differentiating HCC from cirrhosis can be found in Supplementary File 5. Notably, 164 genes were consistently differentially expressed across all three datasets, highlighting their potential as robust biomarkers for distinguishing HCC from cirrhosis (as detailed in Supplementary Table S4). Fig. 6 presents a Venn diagram illustrating the overlap of DEGs among these datasets, emphasizing the genes consistently associated with HCC in comparison to cirrhosis.

### 3.5 Etiology-agnostic DEG signature identifies 89 Pan-HCC therapeutic targets

We identified DEGs that are consistently expressed differentially in HCC tissue compared to other liver conditions, 41, 3568 DEGs (1695 upregulated vs. 1873 downregulated) distinguishing HCC from HCV-related cirrhosis, and 4403 DEGs (2539 upregulated vs. 1864 downregulated) distinguishing HCC from chronic hepatitis of various etiologies. Furthermore, we identified 939 DEGs (232 upregulated vs. 707 downregulated) differentiating HBV-related HCC from HBV-related cirrhosis. Comprehensive details of these DEGs, including their expression profiles across the analyzed datasets, are provided in Supplementary File 6.

### 3.6 Protein-protein interaction networks

**Fig. 7:**
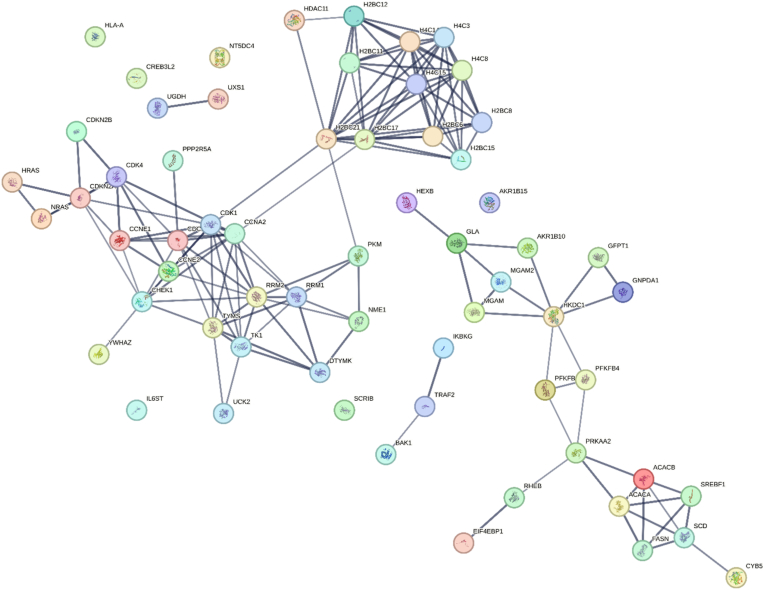
PPI network illustrating interplay among various proteins encoded by DEGs, revealing key hubs and interaction clusters that may play critical roles in HCC.

Functional enrichment analysis of the 125 conserved DEGs was performed using DAVID, identifying 64 genes significantly enriched (FDR <0.05) in cancer-related pathways, including cell cycle regulation, metabolic processes, and immune response. These enriched DEGs were then analyzed using STRING with a high-confidence interaction score (>0.7) to construct a PPI network. The resulting network, as illustrated in Fig. 7, highlights the complex interplay among various proteins encoded by DEGs, revealing key hubs and interaction clusters that may play critical roles in HCC pathogenesis. The PPI network analysis revealed several prominent clusters of interacting proteins, indicating potential pathways and biological processes that are significantly altered in HCC.

Notably, several central nodes, including cyclin-dependent kinase 1 (*CDK1*), cyclin E1 (*CCNE1*), and thymidylate synthase (*TYMS*), were identified as major hubs within the network. These proteins are known to be involved in cell cycle regulation, DNA replication, and repair, processes that are frequently dysregulated in cancer, suggesting their pivotal role in HCC development. Furthermore, the interaction network displayed extensive connectivity among proteins related to metabolic pathways, particularly those involved in glycolysis and lipid metabolism. Key enzymes such as hexokinase 2 (*HK2*), pyruvate kinase M1/2 (*PKM*), and acetyl-CoA carboxylase alpha (*ACACA*) were highlighted, reflecting the metabolic reprogramming often observed in cancer cells, known as the Warburg effect. This metabolic shift may contribute to the aggressive nature of HCC and its resistance to conventional therapies.

Interestingly, the network also emphasized interactions involving proteins associated with cellular stress responses and immune modulation, such as *CAT*, superoxide dismutase 1 (*SOD1*), and interleukin-6 receptor (*IL6R*). The dysregulation of these proteins could be linked to the inflammatory microenvironment commonly observed in HCC, especially in the context of underlying liver conditions like cirrhosis and hepatitis. PPI network analysis of DEGs in HCC compared to other liver conditions has uncovered critical molecular interactions that could serve as potential biomarkers or therapeutic targets. The identified hubs and clusters within the network offer insights into the underlying mechanisms of HCC pathogenesis and highlight the complexity of its molecular landscape. Further investigation into these interactions and their functional implications may provide valuable avenues for the development of targeted therapies and improve the clinical management of HCC.

### 3.7 Hub gene validation and overall survival

To assess the clinical relevance of PPI network hubs, we analyzed overall survival (OS) and disease-free survival (DFS) using TCGA-LIHC data (n = 374 HCC patients). Accordingly, high expression of cell cycle hubs (*CDK1*, *CCNE1*) correlated with shorter OS and poor prognosis (log-rank *p* < 0.001, HR = 1.8) and DFS (*p* = 0.003, HR = 1.5). Moreover, *PKM* upregulation was linked to advanced tumor stage (T3/T4, *p* = 0.02) and vascular invasion (*p* = 0.01). Finally, *CAT* (antioxidant) overexpression was associated with longer OS in HBV-HCC, and hence, showed a protective effect (*p* = 0.04, HR = 0.7).

## 4 Discussion

This study undertook a comprehensive multi-database analysis to identify crucial DEGs associated with HCC that differentiate it not only from normal liver tissue but also from other liver conditions such as cirrhosis and viral hepatitis. Our findings suggest that these DEGs have strong potential to serve as independent prognostic biomarkers for HCC patients. By integrating data from 19 distinct dataset groups, encompassing various comparisons between HCC and other liver conditions, we were able to identify a robust set of DEGs consistently distinguishing HCC from both normal and pathological liver tissues. These DEGs are thoroughly documented in Supplementary Files 2-6, providing a valuable resource for researchers seeking to identify potential biomarkers for further experimental validation.

The differential gene expression (DGE) analysis comparing HCC tissues to normal liver tissues across five datasets highlighted a substantial number of DEGs, reflecting the extensive molecular alterations that occur in HCC. Among these, 125 genes were consistently differentially expressed across all datasets, suggesting their utility as universal biomarkers for HCC. These genes play critical roles in various fundamental cellular processes, including cell cycle regulation and mitosis; signal transduction and cellular communication; metabolic pathways such as amino acid metabolism, fatty acid oxidation, glycolysis, and drug metabolism; ion, nutrient, and metabolite transport across cellular membranes; and cellular stress responses, including oxidative stress and damage repair. Particularly, genes related to retinol and retinoid metabolic processes, such as *CYP2C9*, *CYP2E1*, *CYP39A1*, *CYP3A4*, *CYP4A11*, *CYP4F2*, *CYP8B1*, *RDH5*, and *RDH16*, were significantly enriched among the common DEGs. Previous studies have linked altered retinol homeostasis to various liver diseases, including non-alcoholic fatty liver disease (NAFLD) and NASH, which can progress to hepatic fibrosis and eventually HCC [32,33].

Additionally, members of the solute-carrier (SLC) gene superfamily, including *SLC22A1*, *SLC27A5*, *SLC38A4*, and *SLCO1B3*, were identified as important common DEGs. The SLC gene superfamily has been implicated in tumorigenesis, metastasis, and chemoresistance in HCC, suggesting that the dysregulation of these genes may offer novel strategies for HCC diagnosis [34,35]. Moreover, SLC genes have demonstrated promising accuracy and generalizability in assessing prognosis and predicting survival outcomes for HCC patients [34].

Despite the abundance of research on HCC biomarkers, particularly in the context of HBV-related HCC, there remains a gap in the comprehensive exploration of prognostic biomarkers for this subtype of the disease. Our analysis comparing HCC HBV-infected tissues with non-cancerous HBV-infected liver tissues identified 14 genes consistently differentially expressed across all seven datasets, highlighting their potential as specific biomarkers for HCC in the context of HBV infection. These genes include *ABCA8*, *CAT*, *CCL20*, *F11*, GABARAPL1, *GADD45B*, *GCH1*, *MT1G*, *MT1M*, *RCAN1*, *RND3*, *S100P*, *SOCS2*, and *ZFP36*. Particularly, *ABCA8* and *GADD45B* emerged as prominent candidates, indicating their significant role in distinguishing HCC from non-cancerous HBV-infected tissues. Other studies have identified prognostic-related genes in HBV-associated HCC, such as *TYMS*, *MAD2L1*, *CCNA2*, *CDK1*, and *SPP1*, with some of these genes also emerging as common DEGs in our analysis [36]. Moreover, research by Zeng et al. identified *KIF11*, *TPX2*, *KIF20A*, and *CCNB2* as potential independent prognostic genes and diagnostic targets for HBV-related HCC [37].

Notably, hub genes like *CDK1* and *CCNE1* not only dominated network topology but also predicted poor survival in independent cohorts, reinforcing their role as therapeutic targets. Conversely, the association of *CAT* with improved OS in HBV-HCC suggests etiology-specific protective mechanisms, possibly via oxidative stress mitigation. These findings align with prior studies implicating *CDK1* in sorafenib resistance and *PKM* in metabolic reprogramming [38], but highlight the need for etiology-stratified survival analyses. These findings suggest that the development and progression of HBV-related HCC are closely linked to pathways involving cell cycle regulation, mitosis, p53 signaling, retinol metabolism, and organic acid catabolism.

Similarly, the comparison of HCC HCV-infected tissues with non-cancerous HCV-infected liver tissues revealed 221 DEGs consistently differentially expressed across all four datasets, underscoring their robustness as biomarkers for HCC in HCV-infected patients. Previous studies, such as those by Zhang et al., have reported significant upregulation of cell cycle-related genes in HCV-related HCC, including *CDK1*, *CCNB1*, *CDC20*, *NEK2*, *AURKA*, *RACGAP1*, *CDKN2A*, *CDKN2B*, *CDKN3*, *RRM2*, and *ASPM* [39]. Liu et al. further identified 368 DEGs and 10 hub genes as potential diagnostic biomarkers and therapeutic targets for HCV-related HCC, highlighting *CCNB1*, *KIF20A*, and *HMMR* as candidate targets for diagnosis and therapy [40].

It has been described that there is a potential molecular contributor to the well-documented sex disparity in HCC incidence (male: female ratio ∼3:1) [41]. Although sex-specific analysis was not a primary focus, our results identified multiple sex-associated DEGs within the core 125-gene signature, including: (1) the androgen-regulated gene *CYP3A4* (upregulated in HCC and involved in androgen metabolism), and (2) the X chromosome-encoded histone modifier *UTX/KDM6A* (frequently altered in male-predominant HCC) [42].

While alpha-fetoprotein (AFP) remains the most widely used serum biomarker for HCC in clinical practice, its diagnostic limitations are well-documented. Current data indicate AFP demonstrates suboptimal sensitivity (∼60 %) and specificity (∼80 %), with particular shortcomings in detecting early-stage HCC and distinguishing malignant lesions from benign liver conditions such as cirrhosis or chronic hepatitis [43]. These limitations frequently lead to both false-negative results in AFP-negative tumors and false-positive elevations in non-malignant liver disease, underscoring the critical need for more reliable biomarkers. Our study identifies promising biomarker candidates that could address these limitations through three key mechanisms: (1) detection of AFP-negative HCC cases (e.g., via ABCA8 in HBV-associated HCC), (2) etiology-specific patient stratification, and (3) identification of therapeutic targets (e.g., CCNE1-CDK2 pathway inhibitors). Notably, metabolic DEGs such as *DMGDH* show particular potential for integration into emerging multi-analyte panels like the GALAD score. The robust DEG signatures and protein interaction networks revealed in our analysis provide a strong foundation for developing next-generation HCC diagnostic tools and targeted therapies. The robustness of certain genes and proteins across multiple datasets emphasizes their potential as diagnostic and prognostic tools in HCC. Moreover, understanding the molecular interactions and pathways involved in HCC could pave the way for targeted therapies aimed at disrupting specific pathways altered in this malignancy. Further functional validation of these biomarkers and therapeutic targets is essential to translate these findings into clinical practice and improve patient outcomes.

While our meta-analysis provides a robust transcriptomic landscape of HCC across etiologies, we acknowledge several limitations. Most critically, the absence of experimental validation reflects the computational nature of this study, which prioritized large-scale pattern recognition across 19 heterogeneous datasets over mechanistic investigation. Specifically, functional validation of our core signatures—particularly the 125 pan-etiology DEGs and 220 HCV-specific DEGs—would require: (1) multi-omics profiling (RNA-seq/proteomics) in etiology-stratified patient cohorts to confirm cross-platform consistency (2) CRISPR-based screens in HBV+ (e.g., HepG2.2.15), and HCV+ (e.g., JFH-1-infected Huh7) HCC cell lines to establish causality for hub genes like *CDK1* and *ABCA8*, and (3) longitudinal studies in preclinical models that replicate the cirrhosis-to-HCC transition (6–18 months). For clinical translation, three validation tiers are essential: First, analytical validation via qPCR/Nanostring in retrospective HCC tissue banks should assess technical reproducibility of top candidates (e.g., *CYP2C9* for metabolic dysfunction, *GADD45B* for HBV-specific progression). Second, functional studies using patient-derived organoids must test whether targeting these genes alters tumorigenic phenotypes (proliferation, invasion). Finally, prospective multicenter trials should evaluate liquid biopsy applications, particularly for the 14-gene HBV signature (*ABCA8*, *S100P*) and 164 cirrhosis-to-HCC transition markers, benchmarked against current standards like AFP.

These validations would address key gaps: confirming etiology-specific biomarker performance in early-stage HCC, elucidating whether identified DEGs are drivers or bystanders, and determining their utility in non-invasive diagnostics. Despite these limitations, our conserved signatures provide a high-confidence foundation for such studies, with particular translational promise for metabolic (*SLC22A1*) and cell-cycle (*CCNE1*) targets in HBV/HCV subgroups.

## 5 Conclusion

Our comprehensive DGE analysis and PPI network construction provide a detailed molecular landscape of HCC, offering valuable insights into its pathogenesis and potential therapeutic intervention points. These findings will inform researchers and clinicians in identifying potential biomarkers for the diagnosis and prognosis of HCC, distinguishing it from other liver conditions, and evaluating patient outcomes from chronic hepatitis, liver fibrosis, or cirrhosis. Future research should focus on validating these findings in clinical settings and exploring the functional roles of the identified genes and proteins in HCC progression and treatment.

## CRediT authorship contribution statement

**Babak Khorsand:** Visualization, Software, Methodology, Investigation, Formal analysis, Data curation, Conceptualization. **Nazanin Naderi:** Investigation, Methodology. **Seyedeh Sara Karimian:** Formal analysis, Investigation. **Maedeh Mohaghegh:** Formal analysis, Software. **Alireza Aghaahmadi:** Data curation, Formal analysis. **Seyedeh Negin Hadisadegh:** Investigation. **Mina Owrang:** Writing – original draft. **Hamidreza Houri:** Conceptualization, Data curation, Funding acquisition, Project administration, Supervision, Validation, Writing – review & editing.

## Ethics approval

Not applicable.

## Clinical trial number

Not applicable.

## Consent for publication

Not applicable.

## Availability of data and materials

The data that support the findings of this study are available in the article and related supplementary files.

## Funding

Financial support for this study was provided by the Celiac Disease and Gluten Related Disorders Research Center, Research Institute for Gastroenterology and Liver Diseases, Shahid Beheshti University of Medical Sciences, Tehran, Iran, under grant number IR.SBMU.RETECH.REC.1404.080.

## Declaration of competing interest

The authors declare that they have no known competing financial interests or personal relationships that could have appeared to influence the work reported in this paper.

## Data Availability

The data that support the findings of this study are available in the article and related supplementary files.
