# Supplementary material for: Comprehensive transcriptomic analysis of hepatocellular Carcinoma: Uncovering shared and unique molecular signatures across diverse etiologies
Source: Biochem Biophys Rep. 2025 Jun 30;43:102123. doi: 10.1016/j.bbrep.2025.102123 (PMC12269975; doi:10.1016/j.bbrep.2025.102123)
Supplement: Multimedia component 1 [file mmc1.pdf]

Comprehensive Transcriptomic Analysis of Hepatocellular Carcinoma: Uncovering Shared and Unique Molecular Signatures Across Diverse Etiologies

Babak Khorsand, Nazanin Naderi, Seyedeh Sara Karimian, Maedeh Mohaghegh, Alireza Aghaahmadi, Seyedeh Negin Hadisadegh, Mina Owrang, Hamidreza Houri

Correspondence: Hamidreza Houri, Foodborne and Waterborne Diseases Research Center, Research Institute for Gastroenterology and Liver Diseases, Shahid Beheshti University of Medical Sciences, Shahid Arabi Ave., Yemen St., Velenjak, Tehran, Iran. Email: hr.houri@sbmu.ac.ir

Supplementary Tables

Supplementary Table S1. Core 125-gene pan-etiology HCC signature shared across five datasets.

Pan-Etiology HCC Signature (DEGs in HCC vs. Normal Liver)			
**Upregulated DEGs in HCC (Positive log2FC)**	**Function (Pathway) (Typically involved in oncogenic processes)**	**Downregulated DEGs in HCC (Negative log2FC)**	**Function (Pathway) (Often tumor suppressors or metabolic enzymes)**
ABCA8	Cholesterol transport, lipid metabolism	***ADGRG7***	G-protein coupled receptor activity
ACADS	Fatty acid oxidation	***AKR1B10***	Detoxification, lipid metabolism
ACSL1	Fatty acid metabolism	***ANXA10***	Calcium-binding, cell adhesion
ACSM3	Fatty acid activation	***ASPM***	Mitotic spindle formation, cell proliferation
ACSM5	Fatty acid metabolism	***BSG***	Extracellular matrix remodeling
ADH4	Alcohol metabolism	***CCT3***	Chaperonin, protein folding
AKR1D1	Bile acid synthesis	***CPED1***	Unknown function

ALDH1L1	Folate metabolism	*CSRNP1*	Transcriptional regulation
ALDH6A1	Valine and pyrimidine metabolism	*CXCL2*	Inflammatory response
APOC4	Lipid transport	*ECM1*	Extracellular matrix remodeling
APOF	Lipid metabolism	*EGR1*	Transcription factor, cell proliferation
C6	Complement system activation	*FAM13A*	Unknown function
C7	Complement system activation	*FGB*	Blood coagulation
C8A	Complement system activation	*FGG*	Blood coagulation
CAT	Antioxidant, hydrogen peroxide degradation	*FOS*	Transcription factor, cell proliferation
CETP	Cholesterol ester transfer	*FOSB*	Transcription factor, stress response
CFHR3	Complement regulation	*GABARAPL1*	Autophagy
CLEC1B	Immune response, platelet activation	*GADD45B*	DNA repair, stress response
COLEC11	Immune response, collagen binding	*GPC3*	Cell growth regulation
CPEB3	mRNA translation regulation	*LAPTM4B*	Lysosomal function, oncogenesis
CPS1	Urea cycle, ammonia detoxification	*MCM3*	DNA replication
CTH	Cysteine metabolism	*MCM6*	DNA replication
CXCL12	Chemokine, cell migration	*MDK*	Growth factor, angiogenesis
CYP1A2	Drug metabolism	*MT1E*	Metal ion binding, detoxification
CYP26A1	Retinoic acid metabolism	*MT1G*	Metal ion binding, detoxification
CYP2C9	Drug metabolism	*NNMT*	Nicotinamide metabolism
CYP2E1	Toxin metabolism	*NUSAP1*	Mitotic spindle regulation
CYP39A1	Bile acid synthesis	*PEG10*	Retrotransposon-derived protein, oncogenesis

CYP3A4	Drug metabolism	***PRC1***	Cytokinesis regulation
CYP4A11	Fatty acid oxidation	***SF3B4***	mRNA splicing
CYP4F2	Lipid metabolism	***SKAP1***	Immune cell signaling
CYP8B1	Bile acid synthesis	***SPINK1***	Protease inhibitor
DBH	Neurotransmitter synthesis	***SPP1***	Extracellular matrix remodeling (Osteopontin)
DCN	Extracellular matrix organization	***STEAP3***	Iron transport
DCXR	Ketone and sugar metabolism	***TDO2***	Tryptophan metabolism
DNASE1L3	DNA degradation	***TOP2A***	DNA replication, cell proliferation
EPHX2	Epoxide hydrolysis	***UBD***	Ubiquitin-like protein
FBP1	Gluconeogenesis	***UBE2C***	Ubiquitin conjugation
FCN3	Immune response, pathogen recognition		
FETUB	Bone development, calcium regulation		
FMO3	Drug and toxin metabolism		
FTCD	Histidine metabolism		
FXYD1	Ion transport regulation		
GBA3	Glycoside hydrolysis		
GCDH	Lysine metabolism		
GHR	Growth hormone signaling		
GLS2	Glutamine metabolism		
GLYAT	Glycine metabolism		
GLYATL1	Glycine metabolism		
HAMP	Iron homeostasis		
HAO1	Glycolate metabolism		
HAO2	Fatty acid metabolism		
HGFAC	Hepatocyte growth factor activation		
HOGA1	Mitochondrial metabolism		
HPD	Tyrosine metabolism		
HPR	Haptoglobin binding		
HPX	Heme transport		
INMT	Neurotransmitter metabolism		
KBTBD11	Protein ubiquitination		

KLKB1	Blood coagulation	
KMO	Tryptophan metabolism	
LCAT	Cholesterol esterification	
LECT2	Immune regulation	
LEPR	Leptin receptor, energy metabolism	
LIPC	Lipid metabolism	
NAT2	Drug metabolism	
NDRG2	Cell differentiation	
PAMR1	Extracellular matrix remodeling	
PANK1	Coenzyme A synthesis	
PCK1	Gluconeogenesis	
PDK4	Glucose metabolism	
PLG	Fibrinolysis	
PON3	Detoxification, lipid metabolism	
RCAN1	Calcineurin inhibitor	
RCL1	Ribosome biogenesis	
RDH16	Retinol metabolism	
RDH5	Retinol metabolism	
RNASE4	RNA degradation	
SLC22A1	Organic cation transport	
SLC27A5	Fatty acid transport	
SLC38A4	Amino acid transport	
SLCO1B3	Bile acid transport	
SOCS2	JAK-STAT signaling inhibitor	
SPP2	Extracellular matrix protein	
SRD5A2	Steroid metabolism	
UGT2B10	Drug and steroid metabolism	
VIPR1	Vasoactive intestinal peptide receptor	

Supplementary Table S2. Differentially expressed genes (DEGs) in HBV-associated HCC vs. non-cancerous HBV-infected liver tissue across seven datasets.

**HBV-Specific HCC Signature (DEGs in HBV-HCC vs. HBV-NonTumor)**			
**Upregulated DEGs in HCC (Positive log2FC)**	**Function (Pathway) (Typically involved in oncogenic processes)**	**Downregulated DEGs in HCC (Negative log2FC)**	**Function (Pathway) (Often tumor suppressors or metabolic enzymes)**
CCL20	Chemokine (inflammatory response, immune recruitment)	***ABCA8***	Lipid transport (cholesterol homeostasis)
GABARAPL1	Autophagy (tumor cell survival, stress adaptation)	***CAT***	Catalase (antioxidant, ROS detoxification)
GADD45B	DNA damage response (cell cycle arrest, apoptosis)	***F11***	Coagulation factor XI (blood clotting cascade)
MT1G	Metallothionein (metal ion binding, oxidative stress resistance)	***GCH1***	GTP cyclohydrolase (tetrahydrobiopterin synthesis, nitric oxide regulation)
MT1M	Metallothionein (detoxification, cancer progression)	***RCAN1***	Calcineurin inhibitor (cardiac hypertrophy, stress response)
S100P	Calcium-binding protein (cell proliferation, metastasis)	***RND3***	Rho GTPase (cytoskeleton regulation, cell migration)
ZFP36	mRNA destabilization (anti-inflammatory, tumor suppressor)	***SOCS2***	JAK-STAT signaling inhibitor (growth regulation)

Supplementary Table S3. Differentially expressed genes (DEGs) in HCV-associated HCC vs. non-cancerous HCV-infected liver tissue across four datasets.

**HCV-Specific HCC Signature (DEGs in HCV-HCC vs. HCV-NonTumor)**			
**Upregulated DEGs in HCC (Positive log2FC)**	**Function (Pathway) (Typically involved in oncogenic processes)**	**Downregulated DEGs in HCC (Negative log2FC)**	**Function (Pathway) (Often tumor suppressors or metabolic enzymes)**
ANLN	Cytokinesis, cell proliferation	***ABCA8***	Lipid transport
ASPM	Mitotic spindle organization	***ABCA9***	Lipid homeostasis
AURKA	Mitotic kinase, centrosome maturation	***ACAA2***	Fatty acid oxidation
BIRC5	Apoptosis inhibition (Survivin)	***ACADSB***	Branched-chain amino acid metabolism
BUB1	Spindle assembly checkpoint	***ACSM3***	Fatty acid metabolism
BUB1B	Chromosome segregation	***ADAMTS13***	Von Willebrand factor cleavage
CCNA2	Cell cycle G1/S transition	***ADGRG7***	G protein-coupled receptor
CCNB1	Cyclin-dependent kinase regulator	***AGXT2***	Glyoxylate detoxification
CCNB2	Mitotic cyclin	***ANGPTL6***	Angiopoietin-like protein
CCNE2	G1/S phase transition	***ASPA***	Aspartoacylase (brain metabolism)
CDC20	Anaphase-promoting complex	***ASS1***	Urea cycle (argininosuccinate synthase)

CDC25C	Cell cycle progression (G2/M)	*AVPR1A*	Vasopressin receptor
CDC6	DNA replication initiation	*BCAT1*	Branched-chain amino acid metabolism
CDCA3	Cell cycle regulator	*BCO2*	Carotenoid metabolism
CDCA5	Sister chromatid cohesion	*BDH2*	Ketone body metabolism
CDCA8	Chromosome passenger complex	*C1R*	Complement system
CDHR2	Cell adhesion	*C1RL*	Complement activation
CDK1	Mitotic entry and progression	*C3P1*	Unknown function
CENPE	Kinetochore function	*C4orf46*	Unknown function
CENPF	Chromosome segregation	*C7*	Complement component
CENPK	Centromere assembly	*C9*	Complement component
CENPW	Centromere protein	*CAP2*	Actin binding
CEP55	Cytokinesis regulator	*CCL19*	Chemokine (immune recruitment)
CHEK1	DNA damage response	*CD109*	TGF-β signaling
CKAP2L	Spindle organization	*CD200*	Immune regulation
DEPDC1	Cell proliferation	*CD5L*	Apoptosis regulation
DEPDC1B	GTPase regulation	*CDKN2B*	Cell cycle inhibitor (p15)
DLGAP5	Spindle pole integrity	*CDKN3*	Cyclin-dependent kinase inhibitor
DTL	DNA damage response	*CETP*	Cholesterol ester transfer
E2F7	Transcription factor	*CFHR3*	Complement regulation
E2F8	Cell cycle regulation	*CFP*	Complement factor
ECT2	Rho GTPase activator	*CLEC1B*	C-type lectin
ENAH	Actin cytoskeleton remodeling	*CLEC4G*	C-type lectin (immune defense)

FAM83D	Wnt/β-catenin signaling	***CLEC4M***	Pathogen recognition
FANCD2	DNA repair	***COLEC10***	Collectin (innate immunity)
GPC3	Glypican-3 (HCC biomarker)	***CRHBP***	Corticotropin-binding protein
GTSE1	Cell cycle progression	***CRNDE***	Long non-coding RNA
HMMR	Hyaluronan-mediated motility	***CXCL14***	Chemokine
KIF11	Mitotic spindle motor protein	***CYP1A2***	Drug metabolism (P450)
KIF14	Cytokinesis regulation	***CYP2B6***	Xenobiotic detoxification
KIF15	Microtubule motor protein	***CYP2C8***	Drug metabolism
KIF18B	Chromosome alignment	***CYP2C9***	Warfarin metabolism
KIF20A	Vesicle trafficking	***CYP3A4***	Steroid/drug metabolism
KIF23	Cytokinesis	***CYP4A11***	Fatty acid ω-hydroxylation
KIF2C	Microtubule depolymerization	***CYP4V2***	Fatty acid metabolism
KIF4A	Chromosome condensation	***DEPDC7***	Unknown function
KPNA2	Nuclear import	***DHODH***	Pyrimidine synthesis
MAD2L1	Spindle checkpoint	***DIAPH3***	Actin polymerization
MCM10	DNA replication	***DIRAS3***	Ras GTPase (tumor suppressor)
MELK	Stem cell proliferation	***DLG5***	Cell polarity
MKI67	Proliferation marker	***DNAJC12***	Chaperone protein
NCAPG	Chromosome condensation	***DNAJC6***	Clathrin uncoating
NCAPH	Chromosome cohesion	***DNASE1L3***	DNA degradation (tumor suppressor)
NDC80	Kinetochore assembly	***DUXAP10***	Long non-coding RNA
NEK2	Centrosome duplication	***EBF1***	Transcription factor
NUF2	Kinetochore function	***EDIL3***	Angiogenesis
NUSAP1	Spindle microtubule binding	***ENO3***	Glycolysis
PBK	Mitotic kinase	***ESR1***	Estrogen receptor
PLAC8	Cell proliferation	***FAXDC2***	Fatty acid hydroxylase
PRC1	Microtubule organization	***FBP1***	Gluconeogenesis (frequently lost in HCC)
PTTG1	Securin (chromosome stability)	***FCGR2B***	Fc gamma receptor
RACGAP1	Cytokinesis regulator	***FCN2***	Pathogen recognition (ficolin-2)
RAD51	DNA repair	***FCN3***	Immune response
RAD51AP1	Homologous recombination	***FGA***	Fibrinogen alpha chain
RRM2	DNA synthesis (ribonucleotide reductase)	***FLT1***	VEGF receptor
SKA1	Spindle attachment	***FOSB***	Transcription factor

SPDL1	Mitotic regulation	***FREM2***	Extracellular matrix
STIL	Centriole duplication	***GCDH***	Glutaryl-CoA dehydrogenase
TCF19	Cell cycle regulation	***GPM6A***	Neuronal membrane protein
TOP2A	DNA topoisomerase	***HAMP***	Hepcidin (iron regulation)
TPX2	Spindle assembly	***HBB***	Hemoglobin subunit
TRIP13	Chromosome segregation	***HDAC11***	Histone deacetylase
TTK	Mitotic checkpoint kinase	***HHIP***	Hedgehog signaling inhibitor
UBE2C	Ubiquitin-conjugating enzyme	***IDO2***	Tryptophan catabolism
UBE2T	DNA repair (Fanconi anemia pathway)	***IGF1***	Growth factor
WDHD1	DNA replication	***IGF2BP3***	RNA-binding protein
IGF1	Growth factor signaling	***IGFBP3***	IGF-binding protein
KLF9	Transcriptional regulation	***IGHM***	Immunoglobulin heavy chain
RND3	Rho GTPase regulation	***IL18R1***	Interleukin receptor
SPP1	Osteopontin	***IL1RAP***	Interleukin receptor accessory protein
		ITLN1	Lectin (immune response)
		KANK4	Actin cytoskeleton
		KCND3	Potassium channel
		KLF9	Transcription factor
		LIFR	Leukemia inhibitory factor receptor
		LINC01093	Long non-coding RNA
		LPA	Lipoprotein(a)
		LPL	Lipoprotein lipase
		MARCO	Macrophage scavenger receptor
		MGMT	DNA repair (O6-methylguanine repair)
		MME	Membrane metalloendopeptidase
		MT1F	Metal ion homeostasis
		MT1G	Metal ion homeostasis
		MT1H	Heavy metal detoxification
		MT1M	Metal ion binding
		MT1X	Oxidative stress response
		MT2A	Metal ion binding

	MUC6	Mucin (gastrointestinal protection)
	NAT2	Drug metabolism
	NGFR	Nerve growth factor receptor
	NPY1R	Neuropeptide Y receptor
	OIT3	Oncoprotein-induced transcript 3
	PARPBP	DNA repair
	PHYHD1	Phytanoyl-CoA dioxygenase
	PI15	Protease inhibitor
	PITPNM3	Lipid transfer
	PLCB1	Phospholipase C
	PLIN1	Lipid droplet formation
	PLXNC1	Semaphorin receptor
	PODXL	Cell adhesion
	PRR11	Cell cycle regulation
	PTH1R	Parathyroid hormone receptor
	RBM24	RNA-binding protein
	RCL1	Ribosome biogenesis
	RND3	Rho GTPase regulation
	RNF165	Ubiquitin ligase
	ROBO1	Axon guidance receptor
	SCARA5	Scavenger receptor
	SDS	Serine dehydratase
	SGO2	Chromosome cohesion
	SIGLEC11	Sialic acid-binding lectin
	SKAP1	Immune synapse formation
	SLC38A4	Amino acid transporter
	SMC4	Chromosome condensation
	SMIM24	Small integral membrane protein
	SOCS2	JAK/STAT signaling suppressor
	SPP2	Secreted phosphoprotein
	ST3GAL6	Sialyltransferase
	STAB2	Hyaluronan clearance
	STEAP3	Iron reductase

	STEAP4	Metalloreductase
	TAT	Tyrosine aminotransferase
	TDO2	Tryptophan catabolism
	TIMD4	Immune regulation
	TKT	Pentose phosphate pathway
	TMEM100	Endothelial cell function
	TP53I3	p53-regulated gene
	TRIM16	Protein ubiquitination
	TTC36	Tetratricopeptide repeat protein
	VIPR1	Vasoactive intestinal peptide receptor
	ZFP36	mRNA destabilization
	ESR1	Estrogen receptor

Supplementary Table S4. Differentially Expressed Genes (DEGs) Distinguishing HCC from Cirrhosis.

HCC Early Detection Biomarkers from Cirrhosis (DEGs in HCC vs. Cirrhosis)			
**Upregulated DEGs in HCC (Positive log2FC)**	**Function (Pathway) (Typically involved in oncogenic processes)**	**Downregulated DEGs in HCC (Negative log2FC)**	**Function (Pathway) (Often tumor suppressors or metabolic enzymes)**
A2M	Protease inhibitor (inflammatory response)	***ADH1A***	Ethanol metabolism
AADAT	Kynurenine metabolism (tryptophan catabolism)	***ADH1B***	Xenobiotic clearance
ACSM3	Fatty acid activation (lipid metabolism)	***ADH4***	Retinol metabolism
ADAMTSL2	Extracellular matrix remodeling	***ADH6***	Alcohol detoxification
AFM	Acute-phase response protein	***CYP1A2***	Drug metabolism
AKR7A3	Aflatoxin detoxification	***CYP26A1***	Retinoic acid metabolism
ALDH8A1	Retinaldehyde metabolism	***CYP2A6***	Nicotine metabolism
ALPL	Alkaline phosphatase (bone/liver disease)	***CYP2A7***	Xenobiotic detoxification
ANGPTL6	Angiopoietin-like protein (lipid metabolism)	***CYP2C8***	Drug metabolism
ANXA10	Calcium-binding (HCC biomarker)	***CYP2C9***	Warfarin metabolism

APCS	Pentraxin (innate immunity)	*CYP2E1*	Ethanol/toxin metabolism
APOC4	Lipid transport	*CYP39A1*	Bile acid synthesis
APOF	Lipid metabolism	*F9*	Blood clotting
ASPM	Mitotic spindle (cell proliferation)	*FBP1*	Gluconeogenesis
ASS1	Urea cycle (ammonia detoxification)	*GLS2*	Ammonia detoxification
AURKA	Mitotic kinase (oncogenesis)	*HAMP*	Iron regulation
C1QTNF1	Adipokine signaling	*LCAT*	Cholesterol metabolism
C1R	Complement system activation	*RCAN1*	Stress response
C6	Complement membrane attack	*SLC10A1*	Bile acid uptake
C7	Complement system	*SOCS2*	Growth regulation
CA2	Carbonic anhydrase (pH regulation)	*UGT2B10*	Drug conjugation
CCDC3	Potential tumor suppressor	*VIPR1*	Neuroendocrine signaling
CCL19	Lymphocyte recruitment		
CCL2	Monocyte chemoattractant		
CCL21	Lymphoid organogenesis		
CCL5	T-cell activation		
CCNB2	Cell cycle (G2/M transition)		
CD163	Macrophage scavenger receptor		
CDC20	Anaphase-promoting complex		
CDCA5	Chromosome segregation		
CELSR3	Cell adhesion		
CETP	Cholesterol ester transfer		

CFD	Complement alternative pathway	
CHST4	Glycosaminoglycan biosynthesis	
CLEC1B	Platelet activation	
CLEC4G	Immune response	
COLEC11	Innate immunity	
COX7B2	Mitochondrial respiration	
CRHBP	Stress response	
CRISPLD2	Extracellular matrix	
CXCL12	Hematopoietic stem cell niche	
CXCL14	Immune regulation	
DBH	Neurotransmitter synthesis	
DCN	Extracellular matrix organization	
DNASE1L3	Chromatin clearance	
DPT	Collagen fibrillogenesis	
ECM1	Tumor metastasis	
EGR1	Early growth response	
FBLN5	Elastic fiber assembly	
FCN2	Pathogen recognition	
FCN3	Innate immunity	
FETUB	Bone mineralization	
FGA	Blood coagulation	
FGG	Fibrin clot formation	
FOS	Cell proliferation	
FOSB	Stress response	
GAS1	Growth arrest	
GBA3	Glycolipid metabolism	
GHR	Growth hormone signaling	
GLYAT	Xenobiotic detoxification	
GPC3	HCC biomarker (Wnt signaling)	
GZMK	Immune response	
HAO2	Fatty acid metabolism	
HBA2	Hemoglobin (oxygen transport)	
HBB	Hemoglobin subunit	
HP	Haptoglobin (hemoglobin binding)	

HPX	Heme transport	
HSD17B13	Lipid metabolism	
HSD17B2	Steroid metabolism	
ID1	Cell differentiation	
ID2	Cell cycle regulation	
IFITM1	Antiviral defense	
IGF2BP3	Oncogenic mRNA stabilization	
IGFALS	Growth factor regulation	
IGFBP3	Growth inhibition	
IL7R	Lymphocyte development	
INMT	Neurotransmitter metabolism	
KBTBD11	Protein ubiquitination	
KIF20A	Cytokinesis	
KLRB1	NK cell regulation	
LAMC3	Extracellular matrix	
LEAP2	Antimicrobial peptide	
LHX2	Hepatic differentiation	
LILRB5	Immune regulation	
LPA	Lipoprotein(a)	
LY6E	Immune response	
LYVE1	Lymphangiogenesis	
MARCO	Phagocytosis	
MASP2	Complement activation	
MBL2	Innate immunity	
MCM2	DNA replication	
MDK	Angiogenesis	
MELK	Stem cell maintenance	
MFAP4	Elastic fiber assembly	
MS4A6A	Immune regulation	
MT1A	Metal ion detoxification	
MT1E	Oxidative stress response	
MT1F	Heavy metal binding	
MT1G	Cancer progression	
MT1H	Metal homeostasis	
MT1M	Tumor suppression	

MT1X	ROS scavenging	
MT2A	Zinc ion binding	
MUC13	Epithelial barrier	
NNMT	Metabolic reprogramming	
NUSAP1	Mitotic regulation	
OLFML3	Extracellular matrix	
PBLD	Flavin reductase	
PDGFRA	Cell proliferation	
PEMT	Lipid biosynthesis	
PITX1	Developmental regulation	
PLAC8	Cell proliferation	
PLG	Fibrinolysis	
PLGLB1	Angiogenesis	
PODN	Extracellular matrix	
PRC1	Cytokinesis	
PTGDS	Inflammation	
PTTG1	Chromosome instability	
RBP1	Vitamin A transport	
RDH16	Retinol metabolism	
RDH5	Visual cycle	
RNASE4	RNA degradation	
RND3	Cytoskeleton regulation	
RRAGD	mTOR signaling	
SAA4	Acute-phase response	
SERPINA11	Protease inhibition	
SHBG	Steroid transport	
SLC22A1	Drug uptake	
SLC27A5	Fatty acid transport	
SNORD13	rRNA modification	
SPINT2	Tumor suppression	
SQLE	Cholesterol biosynthesis	
SRPX	Cell adhesion	
STAB2	Hyaluronan clearance	
STEAP3	Iron homeostasis	
TDO2	Immune evasion	
THRSP	Lipid metabolism	

TIMD4	Immune regulation	
TOP2A	DNA replication	
UBE2C	Cell cycle progression	
ZGPAT	Transcriptional repression	
ZIC2	Neural development	
